# Pharmacologic cervical ripening strategies and time-related outcomes: a community hospital cohort study

**DOI:** 10.1016/j.xagr.2026.100658

**Published:** 2026-06-06

**Authors:** Christopher Hayes, Victoria Kucinski

**Affiliations:** Department of Obstetrics and Gynecology, Sisters of Charity Hospital, University at Buffalo, Buffalo, NY

**Keywords:** admit-to-delivery interval, body mass index, Cervidil, Cox proportional hazards, Cytotec, dinoprostone, labor and delivery workflow, labor induction, misoprostol, obstetric workflow, parity, resource utilization, value-based care

## Abstract

**BACKGROUND:**

Pharmacologic cervical ripening is a primary determinant of labor induction efficiency, yet practice-based data from community hospitals remain limited.

**OBJECTIVE:**

To compare time-related outcomes—specifically time to delivery and hospital length of stay—among inductions using misoprostol or dinoprostone, while examining the influence of parity.

**STUDY DESIGN:**

This retrospective cohort study at an urban community hospital (2022–2023) analyzed inductions utilizing only pharmacologic ripening. Exclusion criteria included prolonged antepartum admission, premature rupture of membranes, intrauterine fetal demise, cervical balloon use, or use of both misoprostol and dinoprostone. Patients were categorized by agent: misoprostol (n=188) or dinoprostone (n=477). Primary outcome was time to delivery, with total hospital length of stay also examined. Secondary outcomes included cesarean delivery and neonatal intensive care unit (NICU) admission. Subgroup analyses were performed for nulliparous and multiparous patients.

**RESULTS:**

In the overall cohort, misoprostol was associated with a shorter time to delivery compared with dinoprostone (24.3 vs 28.6 hours; *P*<.001). Total hospital length of stay in hours with misoprostol was also shorter, though not when measured in whole days. Cesarean delivery was observed less frequently among patients receiving misoprostol (14.9% vs 23.5%; *P*=.014), while NICU admission rates did not differ significantly (18.1% vs 15.7%; *P*=.459).

Parity distribution differed between exposure groups however, with a higher proportion of multiparous patients receiving misoprostol. Among nulliparous patients, time to delivery was similar between agents (29.2 vs 30.2 hours; *P*=.363), and differences in cesarean delivery and NICU admission were not statistically significant. Among multiparous patients, misoprostol was associated with a shorter time to delivery compared with dinoprostone (19.7 vs 24.2 hours; *P*=.001), without meaningful differences in cesarean delivery or NICU admission rates.

**CONCLUSION:**

In this community hospital cohort, misoprostol was associated with shorter induction durations than dinoprostone, with efficiency gains primarily driven by outcomes in multiparous patients. These findings suggest that pharmacologic agent selection for cervical ripening can meaningfully influence obstetric workflow.

**AJOG Global Reports at a Glance**

**Why was this study conducted?**

To compare induction-to-delivery times for oral misoprostol vs dinoprostone in a community hospital. To evaluate the influence of parity and practice variation on labor duration and resource utilization.

**Key findings**

Misoprostol significantly reduced time to delivery (24.3 vs 28.6 hours), primarily in multiparous patients. Cesarean delivery and neonatal intensive care unit admission rates did not differ between agents. Total hospital stay in days remained unchanged despite shorter labor courses.

**What does this study add to what is known?**

Provides pragmatic evidence validating misoprostol efficiency within community hospital workflows. Highlights that institutional discharge processes may govern total stay length despite improvements in labor duration.

## Introduction

Cervical ripening is a major determinant of labor induction efficiency and downstream obstetric outcomes.^1^ Two of the most commonly used pharmacologic agents for cervical ripening are misoprostol (Cytotec) and dinoprostone (Cervidil).^1^ In community practice, induction approaches and agent selection may vary by hospital and provider, reflecting local workflow and preference as much as formal protocols.^2^ Randomized trials and recent meta-analyses have reported shorter induction-to-delivery intervals with misoprostol compared with dinoprostone in some settings, although results vary across dosing regimens and populations.^3^

Due to induction duration and total hospital length of stay influencing staffing needs and bed utilization, these metrics are often used as proxies for resource use and cost in obstetric care.^4^ This cost-conscious perspective is particularly relevant in an era of increasing financial pressure on maternity services, including community and rural hospitals.^5^ Practice-based data from community hospitals that link routine cervical ripening choices to time-based outcomes remain comparatively limited, however.^6^

**Figure 1:**
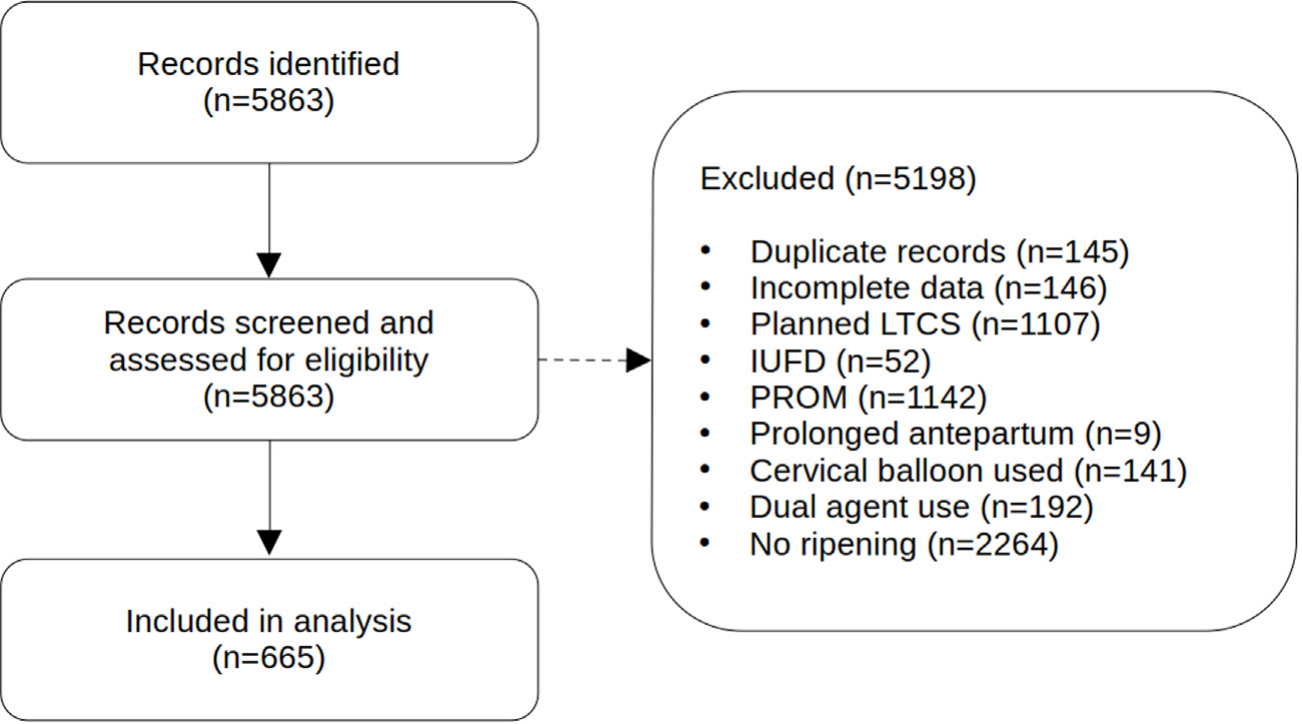
Study flow diagram. Selection of the analytic cohort from electronic medical record–identified deliveries during the study period Abbreviations: LTCS, low transverse cesarean section; IUFD, intrauterine fetal demise; PROM, premature rupture of membranes.

**Figure 2:**
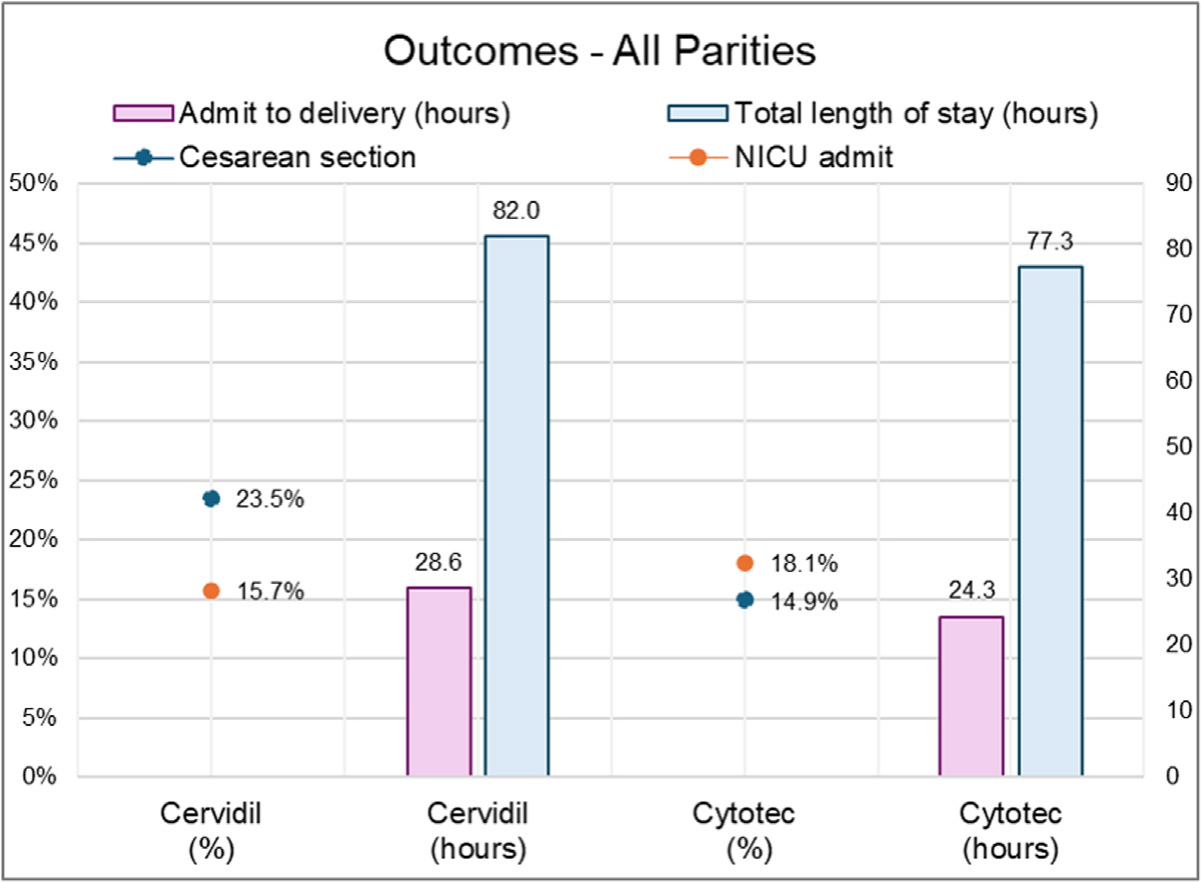
Summary of induction outcomes by cervical ripening strategy in the overall cohort Bars depict median time from admission to delivery and total hospital length of stay (hours), while points depict cesarean delivery and neonatal intensive care unit admission rates.

**Figure 3:**
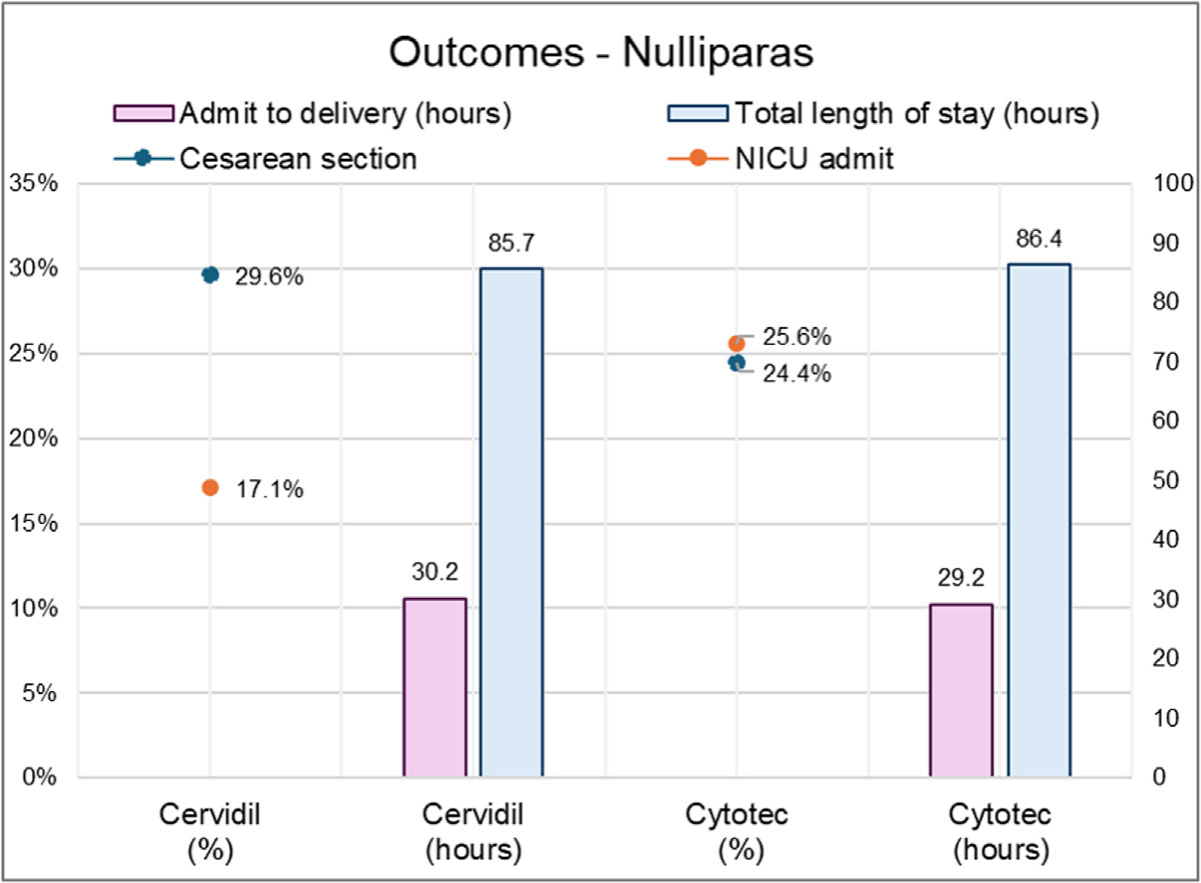
Summary of induction outcomes by cervical ripening strategy among nulliparous patients Bars depict median time from admission to delivery and total hospital length of stay (hours), while points depict cesarean delivery and neonatal intensive care unit admission rates.

**Figure 4:**
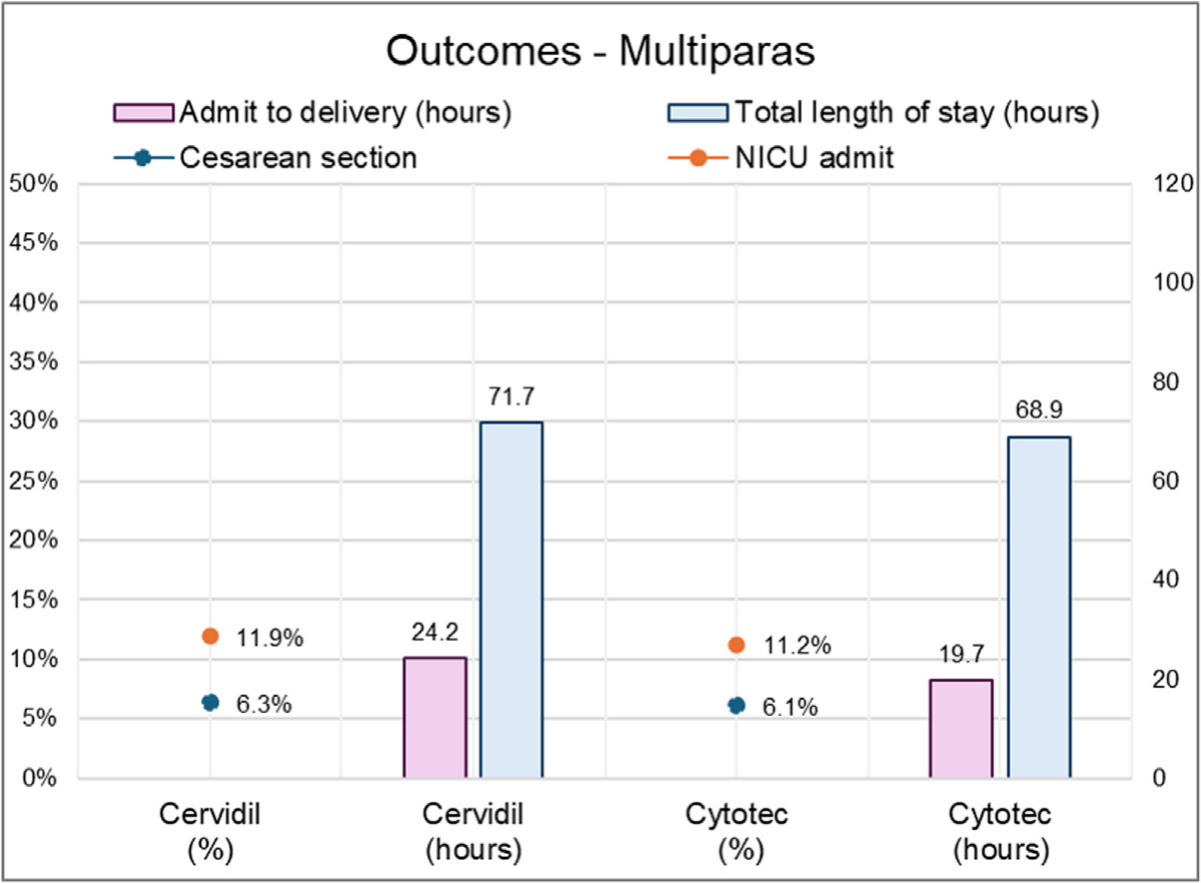
Summary of induction outcomes by cervical ripening strategy among multiparous patients Bars depict median time from admission to delivery and total hospital length of stay (hours), while points depict cesarean delivery and neonatal intensive care unit admission rates.

The objective of this study was to compare time-related outcomes, including time to delivery and total hospital length of stay, among patients undergoing pharmacologic cervical ripening with misoprostol or dinoprostone at a single community hospital. A secondary aim was to examine how parity distribution influenced observed differences in outcomes (Figure 1, Figure 2, Figure 3, Figure 4).

Hayes. Pharmacologic cervical ripening strategies and time-related outcomes: a community hospital cohort study. Am J Obstet Gynecol 2026.

Hayes. Pharmacologic cervical ripening strategies and time-related outcomes: a community hospital cohort study. Am J Obstet Gynecol 2026.

Hayes. Pharmacologic cervical ripening strategies and time-related outcomes: a community hospital cohort study. Am J Obstet Gynecol 2026.

Hayes. Pharmacologic cervical ripening strategies and time-related outcomes: a community hospital cohort study. Am J Obstet Gynecol 2026.

## Methods

### Study design and setting

This retrospective cohort study was conducted at an urban community university-affiliated hospital in Western New York. Electronic medical record data were reviewed for deliveries occurring between January 1, 2022, and December 31, 2023. Institutional review board approval was obtained prior to data extraction.

At this institution, pharmacologic agent selection for cervical ripening is primarily provider driven, as opposed to protocol driven. The resident service, comprising approximately 20% of patients, has an informal preference for misoprostol, whereas private attending physicians typically demonstrate consistent individual preference for a particular pharmacologic ripening strategy, persistent across induction indications. This practice variation mirrors the broader inter-hospital variability reported population-based studies on induction approaches.^2^

Cervical ripening is typically initiated within 1 hour of admission. Any minor delays are unlikely to differ systematically by pharmacologic agent choice. Misoprostol is most often administered orally at a dose of 25 mcg every 2 hours, up to 8 doses.^1^ Dinoprostone is typically placed and removed after 12 hours unless earlier removal is required.^7^ In some cases in our institution, when an initial agent is felt to be insufficient and continued pharmacologic ripening alone is desired, the alternate agent is used sequentially.^8^ These cases have been excluded from analysis in this study, to facilitate a more direct comparison of outcomes.

### Study population

Eligible patients included all deliveries during the study period in which induction of labor required pharmacologic cervical ripening, across all gestational ages. Exclusion criteria included premature rupture of membranes, prolonged antepartum admission (>4 days prior to delivery), intrauterine fetal demise, planned cesarean delivery, cervical balloon use, incomplete or missing data, or sequential use of both misoprostol and dinoprostone.

### Data collection

The dataset was derived directly from the institutional electronic medical record using Epic delivery summary reporting tools. Duplicate records were manually identified and removed. Following export into a spreadsheet for analysis, records with abnormal, incomplete, or out-of-range values were identified through sorting and filtering and subsequently reviewed manually against the electronic medical record. Outcomes were derived from associated standardized fields within the delivery summary report.

### Exposure groups

Patients were categorized into 1 of 2 mutually exclusive groups based on pharmacologic agent employed for cervical ripening: misoprostol only or dinoprostone only.

### Outcomes

The primary outcome was time to delivery. Total hospital length of stay was also examined as a secondary time-related outcome. Both outcomes were defined using admission, delivery, and discharge timestamps recorded in the electronic medical record and analyzed using time-to-event methods, with cesarean delivery treated as a censoring event.^9^ Secondary outcomes included cesarean delivery and neonatal intensive care unit (NICU) admission, the latter serving as a generalized indicator of neonatal clinical concern rather than a direct measure of neonatal morbidity.^10^, ^11^, ^12^

### Statistical analysis

Continuous variables were assessed for normality. Maternal age was summarized using means and standard deviations, while body mass index and time-based variables were summarized using medians and ranges. Group comparisons were performed using analysis of variance or Welch’s t-tests for normally distributed variables and nonparametric tests for non-normally distributed variables. Categorical variables were summarized as counts and percentages and compared using χ² or Fisher’s exact tests.

Time-to-event analyses were restricted to vaginal deliveries, with cesarean delivery treated as a censoring event. Cox proportional hazards regression models were used to estimate crude and adjusted hazard ratios for time to delivery and length of stay, comparing misoprostol with dinoprostone.^13^ Multivariable models adjusted for parity and body mass index.^14^ Statistical significance was defined as a two-sided *P*-value <.05. Analyses were conducted using R statistical software.

## Results

### Patient characteristics

A total of 665 patients met inclusion criteria, of whom 188 received misoprostol only, and 477 received dinoprostone only. Baseline demographic and clinical characteristics for the overall cohort and parity-stratified subgroups are presented in Table 1, Table 2, Table 3.

**Table 1:** Overall cohort characteristics and outcomes

	Cervidil (n=477)	Cytotec (n=188)	*P*-value
Age			.582
Median (Min, Max)	30.00 (17.00, 47.00)	30.00 (18.00, 43.00)	
Mean (SD)	30.24 (5.32)	29.98 (5.33)	
Race			
American Indian or Alaska Native	1 (0.2%)	3 (1.6%)	
Asian	17 (3.6%)	12 (6.4%)	
Asian Indian	18 (3.8%)	5 (2.7%)	
Black or African American	58 (12.2%)	33 (17.6%)	
Multiracial	2 (0.4%)	4 (2.1%)	
Native Hawaiian or Pacific Islander	0 (0.0%)	1 (0.5%)	
Other	28 (5.9%)	18 (9.6%)	
Unknown	2 (0.4%)	1 (0.5%)	
White	351 (73.6%)	111 (59.0%)	
Parity			<.001
Median (Min, Max)	0.00 (0.00, 5.00)	1.00 (0.00, 10.00)	
Mean (SD)	0.40 (0.78)	0.97 (1.33)	
Nulliparous	351 (73.6%)	90 (47.9%)	
BMI			.006
Median (Min, Max)	30.60 (18.60, 65.60)	32.45 (18.50, 65.00)	
Mean (SD)	31.42 (7.20)	33.34 (8.11)	
Gestational age (decimal)			.435
Median (Min, Max)	39.29 (30.71, 41.86)	39.29 (32.86, 41.57)	
Mean (SD)	39.29 (1.34)	39.19 (1.38)	
Delivery mode			.014
Vaginal	365 (76.5%)	160 (85.1%)	
Cesarean	112 (23.5%)	28 (14.9%)	
NICU admit	75 (15.7%)	34 (18.1%)	.459
Neonatal Demise	0 (0.0%)	0 (0.0%)	
Admit to delivery (days)			<.001
Median (Min, Max)	1.13 (0.03, 3.90)	0.89 (0.29, 3.73)	
Mean (SD)	1.19 (0.49)	1.01 (0.48)	
Admit to delivery (hours)			<.001
Median (Min, Max)	27.17 (0.80, 93.53)	21.46 (7.05, 89.42)	
Mean (SD)	28.65 (11.78)	24.27 (11.52)	
Total length of stay (days)			.47
Median (Min, Max)	3.00 (1.00, 8.00)	2.50 (1.00, 7.00)	
Mean (SD)	2.73 (0.99)	2.65 (0.97)	
Total length of stay (hours)			.002
Median (Min, Max)	72.50 (43.78, 211.83)	71.84 (39.02, 169.27)	
Mean (SD)	82.01 (22.51)	77.32 (22.43)

**Table 2:** Nulliparous cohort characteristics and outcomes

	Cervidil (n=351)	Cytotec (n=90)	*P*-value
Age			.06
Median (Min, Max)	30.00 (17.00, 44.00)	28.50 (18.00, 41.00)	
Mean (SD)	29.59 (5.25)	28.41 (5.20)	
Race			.29
American Indian or Alaska Native	0 (0.0%)	1 (1.1%)	
Asian	16 (4.6%)	6 (6.7%)	
Asian Indian	15 (4.3%)	2 (2.2%)	
Black or African American	34 (9.7%)	11 (12.2%)	
Multiracial	2 (0.6%)	1 (1.1%)	
Native Hawaiian or Pacific Islander	0 (0.0%)	0 (0.0%)	
Other	19 (5.4%)	8 (8.9%)	
Unknown	2 (0.6%)	0 (0.0%)	
White	263 (74.9%)	61 (67.8%)	
Parity			
Median (Min, Max)	0.00 (0.00, 0.00)	0.00 (0.00, 0.00)	
Mean (SD)	0.00 (0.00)	0.00 (0.00)	
BMI			.01
Median (Min, Max)	29.90 (18.60, 58.40)	32.75 (18.50, 65.00)	
Mean (SD)	31.00 (7.26)	33.92 (9.04)	
Gestational age (decimal)			.93
Median (Min, Max)	39.43 (33.29, 41.57)	39.57 (34.29, 41.57)	
Mean (SD)	39.40 (1.26)	39.34 (1.48)	
Delivery mode			.33
Vaginal	247 (70.4%)	68 (75.6%)	
Cesarean	104 (29.6%)	22 (24.4%)	
NICU admit	60 (17.1%)	23 (25.6%)	.07
Neonatal Demise	0 (0.0%)	0 (0.0%)	
Admit to delivery (days)			.36
Median (Min, Max)	1.21 (0.35, 3.70)	1.16 (0.35, 3.73)	
Mean (SD)	1.26 (0.47)	1.22 (0.55)	
Admit to delivery (hours)			.36
Median (Min, Max)	29.00 (8.50, 88.85)	27.93 (8.52, 89.42)	
Mean (SD)	30.23 (11.32)	29.24 (13.19)	
Total length of stay (days)			.17
Median (Min, Max)	3.00 (1.00, 8.00)	3.00 (1.00, 7.00)	
Mean (SD)	2.89 (0.99)	3.02 (1.06)	
Total length of stay (hours)			.85
Median (Min, Max)	75.45 (47.57, 211.83)	85.26 (46.05, 169.27)	
Mean (SD)	85.72 (22.56)	86.43 (24.30)

**Table 3:** Multiparous cohort characteristics and outcomes

	Cervidil (n=126)	Cytotec (n=98)	*P*-value
Age			.38
Median (Min, Max)	32.00 (21.00, 47.00)	31.00 (20.00, 43.00)	
Mean (SD)	32.03 (5.13)	31.43 (5.06)	
Race			.02
American Indian or Alaska Native	1 (0.8%)	2 (2.0%)	
Asian	1 (0.8%)	6 (6.1%)	
Asian Indian	3 (2.4%)	3 (3.1%)	
Black or African American	24 (19.0%)	22 (22.4%)	
Multiracial	0 (0.0%)	3 (3.1%)	
Native Hawaiian or Pacific Islander	0 (0.0%)	1 (1.0%)	
Other	9 (7.1%)	10 (10.2%)	
Unknown	0 (0.0%)	1 (1.0%)	
White	88 (69.8%)	50 (51.0%)	
Parity			.02
Median (Min, Max)	1.00 (1.00, 5.00)	2.00 (1.00, 10.00)	
Mean (SD)	1.50 (0.79)	1.86 (1.32)	
BMI			.88
Median (Min, Max)	31.85 (19.30, 65.60)	31.95 (20.40, 53.00)	
Mean (SD)	32.58 (6.95)	32.81 (7.17)	
Gestational age (decimal)			.73
Median (Min, Max)	39.14 (30.71, 41.86)	39.14 (32.86, 41.43)	
Mean (SD)	38.97 (1.52)	39.06 (1.27)	
Delivery mode			.95
Vaginal	118 (93.7%)	92 (93.9%)	
Cesarean	8 (6.3%)	6 (6.1%)	
NICU admit	15 (11.9%)	11 (11.2%)	.88
Neonatal Demise	0 (0.0%)	0 (0.0%)	
Admit to delivery (days)			0
Median (Min, Max)	0.91 (0.03, 3.90)	0.77 (0.29, 1.80)	
Mean (SD)	1.01 (0.50)	0.82 (0.30)	
Admit to delivery (hours)			0
Median (Min, Max)	21.93 (0.80, 93.53)	18.50 (7.05, 43.23)	
Mean (SD)	24.23 (11.93)	19.70 (7.23)	
Total length of stay (days)			.34
Median (Min, Max)	2.00 (1.00, 6.00)	2.00 (1.00, 6.00)	
Mean (SD)	2.28 (0.83)	2.32 (0.73)	
Total length of stay (hours)			.22
Median (Min, Max)	68.50 (43.78, 165.52)	66.97 (39.02, 164.63)	
Mean (SD)	71.67 (18.91)	68.95 (16.76)	

### Overall outcomes

In the overall cohort, misoprostol was associated with a shorter time to delivery compared with dinoprostone (24.3 vs 28.6 hours; *P*<.001; Table 1). Total hospital length of stay measured in hours was modestly shorter with misoprostol (77.3 vs 82.0 hours; *P*=.002), though this difference was not significant when measured in whole days. Cesarean delivery occurred less frequently among patients receiving misoprostol (14.9% vs 23.5%; *P*=.014), though this difference was largely driven by the higher proportion of multiparous patients in the misoprostol group, discussed below in further detail. NICU admission rates did not differ significantly.

Parity distribution differed substantially between groups, with a greater proportion of multiparous patients receiving misoprostol.

### Parity-stratified analyses

Among nulliparous patients, time to delivery was similar between misoprostol and dinoprostone (29.2 vs 30.2 hours; *P*=.363; Table 2). Cesarean delivery rates were numerically lower among patients receiving misoprostol (24.4% vs 29.6%, *P*=.33), while NICU admission rates were numerically higher (25.6% vs 17.1%, *P*=.07); however, neither difference reached statistical significance.

Among multiparous patients, misoprostol was associated with a significantly shorter time to delivery compared with dinoprostone (19.7 vs 24.2 hours; *P*=.001; Table 3). Total hospital length of stay differed minimally between agents (69.0 vs 71.7 hours; *P*=.22), and cesarean delivery (6.1% vs 6.3%; *P*=.95) and NICU admission rates (11.2% vs 11.9%; *P*=.88) were similar.

### Adjusted time-to-event analyses

In Cox proportional hazards models comparing misoprostol with dinoprostone, misoprostol was associated with a significantly higher hazard of delivery in unadjusted analyses (*P*<.001, Table 4). This association persisted after adjustment for parity and body mass index (adjusted *P*<.001), indicating an independent association between misoprostol use and shorter time to delivery.

**Table 4:** Hours to Delivery—Cervidil vs Cytotec, Cox-proportional hazards regression

Characteristic	log(HR)	95% CI	*P*-value
Pharmacologic agent			
Cervidil	—	—	
Cytotec	0.43	0.25, 0.62	<0.001
Characteristic	log(HR)	95% CI	*P*-value
Pharmacologic agent			
Cervidil	—	—	
Cytotec	0.38	0.19, 0.57	<.001
Nulliparous			
FALSE	—	—	
TRUE	−0.95	−1.1, −0.77	<.001
BMI	−0.03	−0.04, −0.02	<.001

Abbreviations: CI, confidence interval; HR, hazard ratio.

In contrast, while misoprostol was associated with shorter total hospital length of stay in unadjusted analyses when measured in hours (*P*=.003, Table 5), this association was no longer statistically significant after adjustment for parity and body mass index (*P*=.2). No significant association between cervical ripening strategy and length of stay was observed when length of stay was measured in whole days in either unadjusted or adjusted models.

**Table 5:** Length of stay (hours)—Cervidil vs Cytotec, cox-proportional hazards regression

Characteristic	log(HR)	95% CI	*P*-value
Pharmacologic agent			
Cervidil	—	—	
Cytotec	0.28	0.10, 0.47	.003
Characteristic	log(HR)	95% CI	*P*-value
Pharmacologic agent			
Cervidil	—	—	
Cytotec	0.12	−0.07, 0.32	.2
Nulliparous			
FALSE	—	—	
TRUE	−1.0	−1.2, −0.81	<.001
BMI	−0.03	−0.04, −0.02	<.001

Abbreviations: CI, confidence interval; HR, hazard ratio.

**Figure 5:**
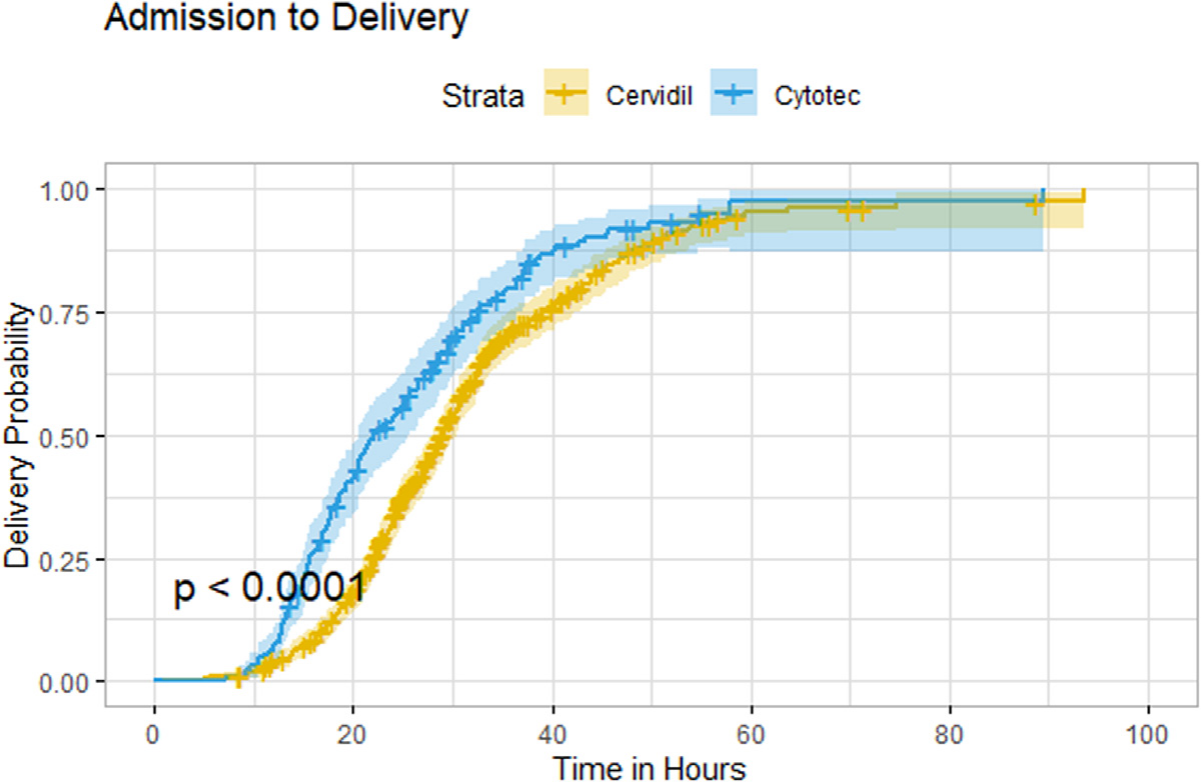
Kaplan–Meier curves depicting time from admission to delivery among patients receiving misoprostol vs dinoprostone for cervical ripening in the overall cohort Vaginal delivery was treated as the event of interest, and cesarean delivery was treated as a censoring event.

**Figure 6:**
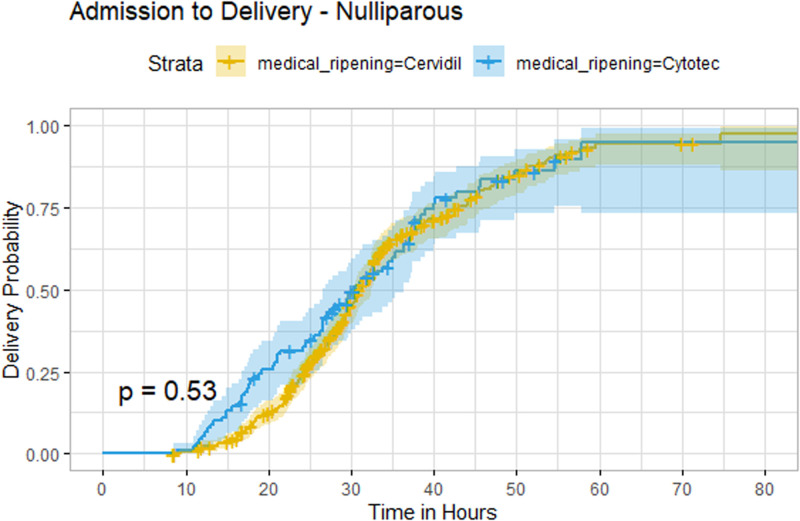
Kaplan–Meier curves depicting time from admission to delivery among nulliparous patients receiving misoprostol vs dinoprostone for cervical ripening Vaginal delivery was treated as the event of interest, and cesarean delivery was treated as a censoring event.

**Figure 7:**
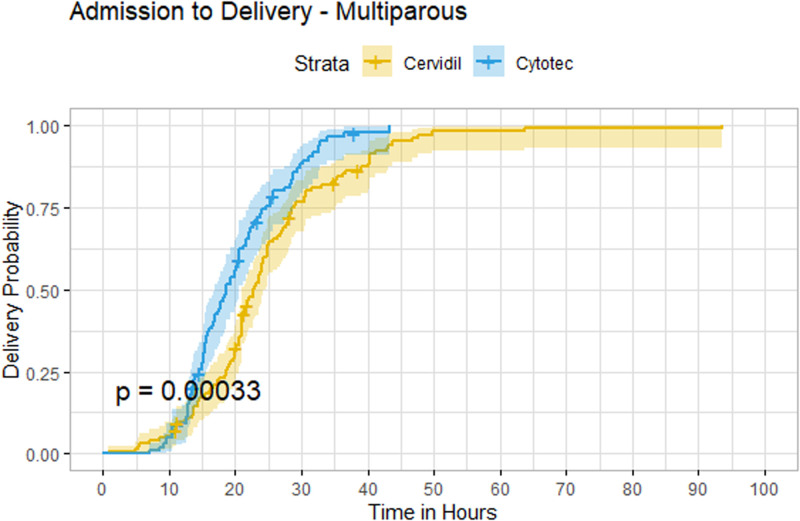
Kaplan–Meier curves depicting time from admission to delivery among multiparous patients receiving misoprostol vs dinoprostone for cervical ripening Vaginal delivery was treated as the event of interest, and cesarean delivery was treated as a censoring event.

These findings are illustrated in Kaplan–Meier curves demonstrating earlier delivery among patients receiving misoprostol compared with dinoprostone (Figure 5, Figure 6, Figure 7). Cesarean delivery was treated as a censoring event, such that patients were followed until vaginal delivery or cesarean, whichever occurred first.

Hayes. Pharmacologic cervical ripening strategies and time-related outcomes: a community hospital cohort study. Am J Obstet Gynecol 2026.

Hayes. Pharmacologic cervical ripening strategies and time-related outcomes: a community hospital cohort study. Am J Obstet Gynecol 2026.

Hayes. Pharmacologic cervical ripening strategies and time-related outcomes: a community hospital cohort study. Am J Obstet Gynecol 2026.

## Discussion

### Principal findings

In this retrospective community hospital cohort, misoprostol use was associated with shorter induction courses compared with dinoprostone, with the magnitude of this association varying by parity. Although overall time to delivery was shorter among patients receiving misoprostol, parity-stratified analyses demonstrated that this difference was driven primarily by outcomes among multiparous patients.

### Results

Among nulliparous patients, time to delivery did not differ meaningfully between pharmacologic agents, and observed differences in cesarean delivery and NICU admission did not reach statistical significance. These findings suggest that residual confounding or underlying differences in patient selection, rather than the pharmacologic agent chosen for cervical ripening itself, may account for observed trends in this subgroup. In contrast, among multiparous patients, misoprostol use was associated with a shorter time to delivery compared with dinoprostone, an association that persisted after adjustment for parity and body mass index. This finding suggests a potential efficiency advantage of misoprostol in this subgroup without evidence of increased cesarean delivery rates or NICU utilization.

This difference is potentially clinically meaningful because labor and delivery time is resource intensive, with associated staffing and cost implications.^4^ Notably, while misoprostol was associated with shorter labor duration, adjusted analyses did not demonstrate an independent reduction in total hospital length of stay. This likely reflects the influence of factors beyond labor duration, i.e. postpartum care practices, discharge processes, etc. on overall hospitalization length.^15^

### Clinical implications

Overall, these results align with prior literature suggesting shorter induction durations with misoprostol,^3^ while extending these observations to a community hospital setting characterized by a mix of provider practices. By focusing on time-related outcomes, this study provides institutionally relevant data that may inform discussions around induction management strategies in similar settings.

### Strengths and limitations

A primary limitation of this study is the absence of standardized Bishop scores or other formal measures of cervical favorability within the delivery records. These data are not systematically linked to delivery summaries in our institutional electronic medical record, precluding adjustment for baseline cervical status without manual chart abstraction.

However, as induction decisions at this hospital follow a binary framework of favorable vs unfavorable cervix, we used the requirement for pharmacologic ripening as a functional clinical surrogate for baseline status. By restricting the analytic cohort to patients requiring such intervention, this framework ensured an unfavorable baseline at the time of treatment initiation, helping to mitigate the impact of unrecorded scores and potential unmeasured variation.

Conversely, a major strength of this research is the utilization of routinely captured electronic medical record data. This accessible approach builds on actual clinical workflows rather than idealized protocols, which enhances reproducibility and facilitates similar analyses at other institutions without requiring additional research personnel or other analytic resources.^16^ While electronic record data possess inherent limitations—including potential entry errors and the truncation of medication lists^17^—we utilized manual filtering and validation of records to ensure the integrity of the findings.

Ultimately, the study provides pragmatic, practice-based evidence that is directly applicable to labor induction management in similar community hospital settings.

### Research implications

Future work would benefit from improved capture of cervical exam data within the electronic medical record. This would ideally take the form of structured fields within the delivery summary to record serial cervical exams, or at minimum the initial exam on patient presentation. Such changes would require collaboration with institutional Epic teams and would be most reasonable to address during planned system upgrades or other periods of workflow redesign. At that same time, other modifications such as preventing truncation of medication lists in the reporting tools, would also be beneficial.

Additional studies could also examine whether observed differences in induction efficiency translate into measurable institutional cost savings or staffing impacts.

## Conclusion

In a community hospital setting, misoprostol use for cervical ripening was associated with shorter induction courses compared with dinoprostone, with the greatest efficiency gains observed among multiparous patients. These findings highlight the potential influence of cervical ripening choice on obstetric workflow and resource utilization in similar practice environments.

## CRediT authorship contribution statement

**Christopher Hayes:** Writing – review & editing, Writing – original draft, Visualization, Validation, Software, Resources, Project administration, Methodology, Investigation, Formal analysis, Data curation, Conceptualization. **Victoria Kucinski:** Writing – review & editing, Validation, Supervision, Resources, Project administration, Methodology, Conceptualization.
